# Developing Trace: A Transdiagnostic Screening Tool for Early Detection of a Pluripotent at‐Risk Mental State

**DOI:** 10.1002/mpr.70046

**Published:** 2025-12-03

**Authors:** Jayne Pickering, Wen Shao, Scott Weich, Caroline F. Dalton, Myles Jones, Chris D. J. Taylor, Markus Reuber, Jessica Kingston, Chris Gibbs, Richard P. Bentall

**Affiliations:** ^1^ University of Sheffield Sheffield UK; ^2^ Sheffield Hallam University Sheffield UK; ^3^ Pennine Care NHS Foundation Trust Ashton‐under‐Lyne UK; ^4^ Royal Holloway University of London Egham UK; ^5^ Cumbria, Northumberland, Tyne and Wear NHS Foundation Trust Newcastle Upon Tyne UK

**Keywords:** at‐risk mental state, borderline personality, hypomania, pluripotent, psychosis, transdiagnostic

## Abstract

**Objectives:**

Early identification of individuals at risk for developing mental health disorders is critical for timely intervention, reducing distress and improving outcomes. This study outlines the development and preliminary validation of ‘TRACE’, a short self‐report measure designed to detect a pluripotent at‐risk mental state (PARMS). The measure captures a range of subthreshold symptoms and traits associated with emerging manifestations of mania, psychosis and borderline personality disorder.

**Methods:**

A sample of 2037 general‐population adolescents and young adults (aged 14–36) completed TRACE questions and other psychometrics.

**Results:**

Exploratory Graph Analysis, undertaken on one‐half of the dataset, generated a three‐factor structure that was successfully replicated in the other half of the data, via confirmatory factor analysis. The final 26‐item scale has acceptable model fit (with a scaled CFI of 0.93, TLI of 0.92, with acceptable‐to‐good error, RMSEA of 0.073) and good concurrent validity with other relevant psychometrics, such as neuroticism and loneliness (*r* = 0.29 and 0.43, respectively).

**Conclusion:**

These findings suggest that the tool may be useful in identifying individuals with a broad, pluripotent vulnerability to develop severe mental health disorders. Future research will focus on validating the tool longitudinally and across diverse populations to assess its predictive utility and clinical value.

## 1 Introduction

In recent decades, researchers have tried to identify individuals who are at risk of developing severe mental health disorders, before symptoms escalate, to improve access to treatment and reduce distress (Correll et al. 2018). One prominent approach to identifying at‐risk individuals is the ‘At‐Risk Mental States’ criteria (ARMS; Yung et al. 1998; Yung and McGorry 1996), which identifies individuals at risk of developing psychosis. Another prominent framework is the ‘Bipolar At‐Risk’ criteria used to identify individuals at risk of developing bipolar I or II disorders (Bechdolf et al. 2010, 2014).

However, emerging evidence challenges the notion that there are separate at‐risk states for different disorders. Five key arguments support a single pluripotent at‐risk state conferring risk across disorders.

First, there are shared genetic markers across schizophrenia, bipolar disorder, major depression, and borderline personality disorder. Polygenic risk scores overlap between these conditions, suggesting a shared biological susceptibility rather than distinct causal pathways for each disorder (e.g., Witt et al. 2017). Heritability studies show children of affected parents are more likely to develop any mental disorder (Rasic et al. 2014).

Second, many symptoms appear across multiple disorders. For example, psychotic symptoms, such as auditory hallucinations, can occur in schizophrenia, bipolar disorder, severe depression and borderline personality disorder. Emotional dysregulation occurs in both bipolar and borderline personality disorders (Miola et al. 2022), and cognitive impairment is common across schizophrenia, bipolar disorder, and major depression (Zhu et al. 2019).

Third, individuals identified as being at risk for one disorder are often later diagnosed with a different condition. For instance, a significant proportion of individuals meeting ARMS criteria for psychosis later develop bipolar disorder or major depression rather than schizophrenia (Hartmann et al. 2021). This is further complicated by symptom change over time (Morgan et al. 2022) and misdiagnoses (e.g., Ayano et al. 2021; Daveney et al. 2019).

Fourth, there are general transdiagnostic risk factors such as early‐life trauma (Hogg et al. 2023; Zhou et al. 2025), loneliness (Nuyen et al. 2020), sleep disturbances (Harvey et al. 2011), urban living (Evans et al. 2023) and socioeconomic disadvantages (Pickett and Wilkinson 2010; Reiss 2013) which are associated with increased risk for several disorders.

Finally, growing research supports a dimensional, rather than categorical, approach to mental health. The HiTOP model (Kotov et al. 2017, 2021; Conway et al. 2019) organises psychopathology hierarchically, from specific symptoms to narrow dimensions, then broad spectra, viewing disorders as overlapping continuous dimensions rather than discrete conditions. These broad underlying factors may represent a single overarching psychopathology or ‘p‐factor’, accounting for shared vulnerability across multiple disorders (Caspi et al. 2014; Caspi and Moffitt 2018). Factor analytic studies suggest that this dimensional approach may offer the best framework for understanding the early stages of severe mental health problems (Lahey et al. 2018).

With these considerations in mind, we advocate for a pluripotent at‐risk mental state (PARMS). The PARMS paradigm acknowledges that early symptoms may indicate a generalised risk for multiple mental‐health conditions, rather than signifying the beginning of a specific trajectory towards a singular disorder. This is the approach taken by Hartmann et al. (2019),  (2021) when developing the Clinical High At‐Risk Mental State (CHARMS) criteria, where they found that 34% of participants transitioned from a non‐specific attenuated syndrome to meeting full diagnostic criteria for either borderline personality disorder, depression, psychosis or bipolar disorder within 12 months (with the majority of conversions being to major depressive disorder). This is a significantly higher conversion rate than typically found using diagnosis‐specific criteria. For example, 34% at 36 months (Fusar‐Poli et al. 2012) or 25% at 36 months (De Pablo et al. 2021).

However, researchers and clinicians need practical tools to identify the PARMS. Existing screening instruments, designed to identify those at risk of specific disorders, do not adequately capture the broad spectrum of symptoms that indicate the onset of severe mental health conditions. Developing and collating separate tools leads to redundancy, incompatible time scales, participant burden, scoring concerns and a lack of (or contradictory) diagnostic cut‐offs. Additionally, tools need to be suitable for younger respondents as the average age for developing a mental health disorder is 14.5, according to a recent meta‐analysis (Solmi et al. 2022). This means that tools need to have an accessible vocabulary and avoid reference to life events mostly associated with older demographics, for example, trouble paying bills. The CHARMS approach uses extensive clinical interviews and long testing batteries. This is excellent for generating rich data for researchers. However, it is too time intensive to be of every‐day clinical use—especially when both time and finances are limited in research and clinical settings. Therefore, there is a need for a short self‐report measure of the pluripotent at‐risk state, suitable for adolescents and young adults; such an instrument could be delivered by non‐experts and would function as a pre‐screener to more thorough clinical evaluation.

Additionally, it is important that we understand the structure of PARMS. That is, whether a general at‐risk state underlies the risk for various disorders, or whether distinct factors correspond to specific outcomes. This has significant implications for both assessment and intervention strategies.

In response to this challenge, we have developed the Transdiagnostic Risk Assessment and Clinical Evaluation (TRACE) self‐report questionnaire. Our questionnaire development followed a Formal Psychological Assessment procedure (Serra et al. 2015), which is a technique for amalgamating questions across multiple measures, cross‐checking with diagnostic criteria and editing out redundancy. This approach takes advantage of the diligent work of previous researchers to ensure that questions are appropriate (e.g., with good face validity, clear and unambiguous, unidimensional, etcetera) while also covering the range of relevant symptomology important to a transdiagnostic approach.

To define a cut‐off for TRACE, we need a criterion variable to correlate with TRACE scores, so we can provisionally establish risk groups. We chose a well‐validated prodromal psychosis measure (Prodromal Questionnaire‐16; Ising et al. 2012). The PQ16 has good to high internal consistency, with Cronbach's alpha of 0.84 in a help‐seeking adolescent study (de Yong et al., 2020) and excellent 1‐month test‐retest reliability (Parabiaghi et al. 2024). Although TRACE aims to predict a pluripotent at‐risk state, rather than psychosis per se, by definition, a pluripotent at‐risk state must include the psychosis at‐risk state. The PQ16 has been shown to correlate with measures of depression and anxiety (Parabiaghi et al. 2024; Long et al. 2024; Nastro et al. 2023; Long et al. 2024) and may be associated with features of borderline personality disorder, albeit with small effect sizes (Long et al. 2024). Some items on the PQ16 such as, ‘I feel uninterested in the things I used to enjoy’, measure symptoms overlapping with depression or general distress. Elevated PQ16 scores might reflect a broader vulnerability or response to psychological distress rather than a specific risk for psychosis. Therefore, the PQ16, while not ideal because it does not include bipolar or borderline risk symptoms, offers a reasonable proxy for identifying a broader transdiagnostic vulnerability to severe mental health disorders.

This paper outlines our steps in developing TRACE and validating its structure within a young population. To understand the factor structure, we perform exploratory analyses with one dataset and confirmatory analyses with a second dataset. In the combined dataset, we then outline the association between TRACE and other relevant psychometric constructs and determine possible cut‐offs for practical use of the TRACE measure. We perform additional analyses to validate TRACE, including measuring its convergent validity (correlation with related psychometrics) and its incremental validity (ability to predict wellbeing scores beyond the PQ16 and relevant demographics). These analyses are essential for illustrating that the tool accurately measures an at‐risk mental state and is suitable as a pre‐screener for research or clinical use.

## 2 Method

### 2.1 Questionnaire Development Process

Our questionnaire development followed a Formal Psychological Assessment (FPA) procedure (Serra et al. 2015). We followed seven developmental steps.

Step 1: we started with a list of 68 prodromal psychosis symptoms identified in a review of at‐risk screening measures by Bernardin et al. (2023).

Step 2: we added 12 (hypo)mania and nine borderline personality disorder features, as identified through the Structured Clinical Interview for DSM‐5 (SCID‐5; First et al. 2015) or the CHARMS criteria (Hartmann et al. 2019). Ten duplicated symptoms were removed, leaving a list of 79 prodromal symptoms. This list was intentionally broad and included fine‐grained and nuanced symptoms.

Step 3: we selected one to three questions addressing each of the 79 symptoms, creating an initial list of 214 questions derived from 25 existing questionnaires (see supplementary materials). We intentionally oversampled questions to select the best fit per symptom.

Step 4: three reviewers independently reviewed the list with an aim to remove any question that was deemed to be poorly phrased, ambiguous, unsuitable for younger respondents, a poor match for that symptom, or too similar to other questions. Questions were rated as keep (2), maybe (1) or remove (0). We retained questions that scored three or more across the three reviewers. After this stage, 136 questions were removed, leaving 78 questions.

Step 5: we compared our list against 30 published prodromal criteria, from the CHARMS and SCID‐5, as well as the Structured Interview for Psychosis (McGlashan et al. 2010), Bipolar‐at‐risk criteria (Bechdolf et al. 2010) and the Comprehensive Assessment of At‐risk Mental Sates (Yung et al. 2005). We added questions if a criterion was not covered and removed questions if they were unrelated to any criteria (i.e., symptoms derived from Bernardin et al. that were too narrow). We removed two criteria, either because they were under‐specified (‘inappropriate sense of humour’) or they were compounded and covered elsewhere (‘transient stress‐related paranoia ideas or severe dissociative symptoms’). After this stage, we had a list of 28 criteria and 71 questions. (Note, this is lower than the original list of 79 criteria because many of the psychosis at‐risk features listed by Bernardin et al. were removed and yet we still met the criteria listed in the above five sets of criteria.)

Step 6: we phrased all questions in the second person, in question format and in simple past tense. We then sought feedback on phrasing and clinical content from clinicians (*n* = 4), a young person without mental health difficulties (*n* = 1) and people with lived experience of mental health problems (*n* = 10, whereby 7 people were in the at‐risk age range, under 35, including two teenagers). Clinicians were given symptoms with questions clustered underneath and were asked to rank the questions in terms of how well they addressed each symptom. The young person (a fifteen‐year‐old female) gave feedback on the appropriateness of the language for young people, and whether the vocabulary and phrasing were appropriate. Those with lived experience of mental health difficulties were given a randomised list of the questions and asked for feedback on the wording, relevance and respectfulness of each question. They rated each question as ‘keep, reword or remove’. All respondents could leave open‐ended feedback. We used the feedback to make minor adjustments to phrasing.

**FIGURE 1:**
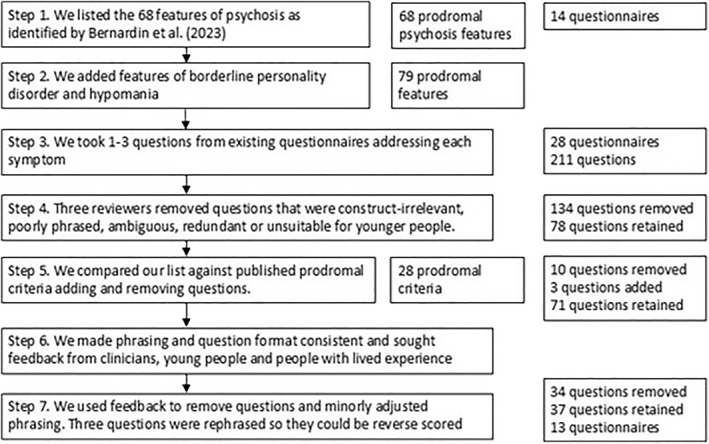
Simplified steps for the TRACE questionnaire development process.

Step 7: using the feedback from step six, we undertook a final round of review, with the aim of limiting each symptom to as few questions as possible. Three reviewers reviewed clinical feedback and removed 34 questions, leaving a final list of 37 questions, derived from 13 questionnaires. Three items were reverse‐coded to address acquiescence bias (Primi et al. 2019). This process is summarised in Figure 1.

### 2.2 Structure Validation Methodology

#### 2.2.1 Participants

Participants were ‘general population’ and were not help‐seeking. A stratified sample of 2037 participants aged 14–36 years (mean 25.3, SD = 6.43) were recruited by the survey company Qualtrics (with 51% female participants). We stratified the sample based on age, gender and UK geographical region (50:50 gender, with age bracket targets of 14–17 (17.3%), 18–24 (27.9%), 25–29 (24.1%) and 30–36 (30.7%) to match the national representation of the UK, and 12 geographical regions). The procedure was approved by the University of Sheffield's School of Psychology Research Ethics Committee (approval number 064323).

#### 2.2.2 Materials

In addition to our questionnaire, we administered the following validated psychometric measures so as to measure the concurrent and discriminant validity of TRACE from other relevant psychometrics.

Depression (Patient Health Questionnaire‐9; Kroenke and Spitzer 2002). An 8‐item version, with answers on a 4‐point Likert scale, asking about symptoms from the past 2 weeks. The suicide item was omitted due to remote testing with vulnerable youth. Scores range from 0–24, and scores ≥ 10 suggest a depressive disorder (Kroenke et al. 2009). In our sample, the internal consistency was good (Cronbach's *α* = 0.89).

Anxiety (Generalized Anxiety Disorder‐7; Spitzer et al. 2006). A 7‐item scale, with answers on a 4‐point Likert scale, asking about symptoms from the past 2 weeks. Scores range from 0–21, and scores ≥ 8 suggest moderate anxiety or ≥ 15 suggest severe anxiety (Plummer et al. 2016). In our sample, the internal consistency was excellent (Cronbach's *α* = 0.92).

Quality of life (Warwick‐Edinburgh Mental Well‐being Scale; Tennant et al. 2007). A 14‐item scale, with answers on a 5‐point Likert scale, asking about well‐being over the past two weeks. Scores range from 14–70, with higher scores indicating better well‐being. In our sample, the internal consistency was excellent (Cronbach's *α* = 0.94).

Sleep quality (Sleep Condition Indicator; Espie et al. 2014). An 8‐item scale, with answers on a 5‐point Likert scale, asking about sleep patterns and quality over the past month. Scores range from 0 to 32, with lower scores indicating more severe sleep disturbance. Scores ≤ 16 may suggest insomnia disorder (Espie et al. 2014). In our sample, the internal consistency was good (Cronbach's *α* = 0.89).

Core‐schemas (Brief Core Schema Scale; Fowler et al. 2006). A 24‐item scale, with binary statement endorsement (yes/no). Endorsed items are followed by a 4‐point Likert scale assessing conviction. The measure has four subscales assessing positive and negative beliefs about the self and others. Higher scores indicate stronger endorsement. Subscales range from 0–24. Internal consistencies were good‐excellent for all subscales: negative self (Cronbach's *α* = 0.83), positive self (Cronbach's *α* = 0.86), negative other (Cronbach's *α* = 0.90) and positive other (Cronbach's *α* = 0.91).

Personality (Big‐Five Inventory‐10; Rammstedt et al. 2013). A 10‐item scale, with answers on a 5‐point Likert scale, measuring the five dimensions of personality (openness, conscientiousness, extraversion, agreeableness, neuroticism). Higher scores reflect greater endorsement of each trait, each subscale ranges from 2–10. Pearson's correlations for four sub‐scales show small to moderate associations for the items: Extraversion *r* = 0.15, *p* < 0.001; Agreeableness *r* = 0.13, *p* < 0.001; Conscientiousness *r* = 0.25, *p* < 0.001 and Neuroticism *r* = 0.33, *p* < 0.001. However, showed poor reliability (*r* = ‐ 0.09, *p* < 0.001).

Psychosis risk (Prodromal Questionnaire‐16; Ising et al. 2012). A 16‐item scale where individuals indicate the presence or absence of psychotic‐like experiences over the past month. Endorsed items are rated for associated distress on a 4‐point Likert scale, with higher total scores suggesting greater risk for psychosis. A score of ≥ 6 positively endorsed questions suggests respondent is at high risk for psychosis (Ising et al. 2012). In our sample, the internal consistency was excellent (Cronbach's *α* = 0.95).

Hopelessness (2‐Item Hopelessness Scale; Everson et al. 1996). A 2‐item scale, with answers on a 5‐point Likert scale. Scores are coded 0–4 then summed, for a score of 0–8. Scores of 6+ are considered highly hopeless. Pearson's correlations indicate a strong association between the items, *r* = 0.61, *p* < 0.001; Loneliness (3‐item Loneliness Scale; Hughes et al. 2004). A three‐item scale, with answers on a three‐point Likert scale (range 3–9). Higher scores reflect greater loneliness. In our sample, the internal consistency was good (Cronbach's *α* = 0.81).

Negative life events (History of Social Punishment Scale; Angelakis et al. 2018; Angelakis and Gooding 2020). An 11‐item scale, with answers on a 5‐point Likert scale, assessing lifetime experiences of social punishment, such as rejection or humiliation. Higher scores indicate greater adversity. In our sample, the internal consistency was good (Cronbach's *α* = 0.89).

We also included a short numerical and verbal fluency measure as a cognitive assessment. Due to the large age range and short duration of the protocol, we could not use standardized psychometrics. Instead, we used a 10‐item find‐the‐synonym test, with 5 options paired with a target word. Based on corpus statistics and prevalence estimates, the words varied from well‐known (e.g., ‘space’) to low prevalence/literary (e.g., ‘cloistered’). We also included three numerical questions, based on percentages and ratios, with a free‐text answer box. In our sample, the internal consistency for our collective cognitive measure was acceptable (Cronbach's *α* = 0.72).

Our piloted 37‐item TRACE scale asked individuals to indicate the presence or absence of experiences over the past month. Endorsed items were rated for associated distress on a 4‐point Likert scale, with higher total scores suggesting greater distress. See supplementary materials for the full pilot questionnaire.

We also collected relevant demographic variables including urbanicity and socioeconomic status and asked about mental health diagnoses of first‐degree relatives.^1^

#### 2.2.3 Procedure

After obtaining consent, we collected basic demographic information (age, sex, ethnicity, SES‐related variables). Participants then completed the measures online and in a set order. Questions were set to compulsory and participants outside of our pre‐specified age range were automatically ejected. The median completion time was 22 min. Participants were reimbursed at Qualtrics' standard rate.

#### 2.2.4 Data‐Cleaning

Datasets that failed our quality‐control checks were removed automatically by Qualtrics, including incomplete or terminated datasets. We included two attention checks, ‘Select ‘agree a little’ for this question’ and ‘Please select the animal from the list below’ (placed in the cognitive assessment). Additionally, we included a sincerity check. To ensure participants gave thoughtful answers, we excluded anybody who failed a speeding check, specified as a completion time under half the median soft‐launch time of 16 min.

## 3 Results^2^

### 3.1 Whole Sample Characteristics

The sample was UK‐based, with 51% female participants, 3% had a trans gender identity, 83% born in the UK, 61% identifying as white British/Irish (no other ethnicity was represented more than 5%) and a stratified age range of 14–36 (*M* = 25, SD = 6). There was a spread of urbanicity and socioeconomic backgrounds, and 48% lived in cities, 29% in towns, 17% in suburbs and 7% in rural locations. Additionally, 46% had a degree or higher‐level qualification and 53% had a mortgage or own their own home. 36% have a first‐degree relative with a diagnosis for a mental health condition (51% unsure, 12% did not).

### 3.2 Stratified Random Data Splitting

The sample was split into two samples via stratified random data splitting, based on age and sex. Sample one (*n* = 1022) had an average age of 25.26 years (SD = 6.89) and were 50.8% female. Sample two (*n* = 1015) had an average age of 25.38 years (SD = 6.36) and were 50.9% female.

### 3.3 Item Rest Corelation

Item rest correlation measures how well each item correlates with the sum scores of all other items; this helps to determine how well each item contributes to the overall construct. Two items had low correlations (< 0.30) and were removed (TR1 and TR4) from the questionnaire and subsequent analyses.

### 3.4 Exploratory Graph Analysis (EGA)—Dataset One

EGA, with polychoric correlations, was conducted to identify the underlying dimensional structure of the questionnaire items, using the remaining 35‐item TRACE scale. The analysis initially generated a three‐community structure. However, the structural consistency and item stability were generally low across 2000 iterated samples. To address this, nine unstable items (replicability < 60%) were excluded. The analysis was repeated with the refined 26‐item measure, leading to significant improvements in structural consistency. The updated consistencies were: 0.96, 0.87, and 0.98 for the three factors. Additionally, all remaining items demonstrated very high stability (replicability > 85%), see Table 1 for detailed results.

**TABLE 1:** Community loadings of the exploratory graph analysis.

Items	Short names	Community 1	Community 2	Community 3
Structural consistency		0.96	0.87	0.98
TR3	Impulsivity	0.99		
TR5	Racing thoughts	1		
TR7	Low mood	1		
TR12	Disorganized thinking	1		
TR15	Emptiness	1		
TR19	Mood cycle	1		
TR21	Irritability	0.99		
TR23	Mood lability	1		
TR24	Disconnected thoughts	1		
TR26	Identity confusion	0.98		
TR27	Poverty of speech	0.97		
TR34	Emotional numbness	1		
TR37	Confused thinking	0.98		
TR6	Auditory hallucinations		1	
TR9	Sound distraction		0.99	
TR17	Reality confusion		0.98	
TR18	Precepted oddity in familiar surroundings		1	
TR29	Pseudo hallucinations		1	
TR30	Suspiciousness		1	
TR33	Paranoia		0.88	
TR13	Pressured speech/talkative			1
TR14	Elevated self‐confidence			1
TR16	Elevated mood or energy			1
TR20	Increased goal directed activities			0.98
TR22	Grandiosity			1
TR25	Idea of reference			1

*Note:* The structural consistency reflects the frequency with which this empirical composition was replicated across all bootstrapped samples *N* = 2000. The value in each community column is the probability that the item was assigned to this community in the bootstrapped samples. For instance, TR5 was assigned to community 1 in all bootstrapped samples.

After considering the clustering of symptoms under each factor, we have named Factor 1 ‘Affective‐cognitive dysregulation’ (or ‘dysregulation’ for short), Factor 2, ‘reality distortion’ and Factor 3, ‘hypomania’.

### 3.5 Confirmatory Factor Analysis (CFA)—Dataset Two

Following EGA, CFAs were performed to test two models: a standard factor model based on the EGA‐derived community structure, and a bifactor model comprising a general factor and three specific factors aligned with the EGA dimensions. CFA was conducted on the refined 26‐item measure to test the fit of the previously identified three‐factor structure. The model was successfully replicated. The model demonstrated acceptable fit, with a scaled Comparative Fit Index of 0.93 and Tucker Lewis Index of 0.92. The Root Mean Square Error of Approximation was 0.073 and the Standardised Root Mean Square Residual was 0.065, both acceptable to good. The Factor correlations were moderate to high: 0.51 (dysregulation and hypomania), 0.72 (reality distortion and hypomania), and 0.79 (dysregulation and reality distortion), suggesting distinct but related factors.

Internal consistency and convergent validity were evaluated using Composite Reliability. These values were high across factors, ranging from 0.84 to 0.88. Additionally, Average Variance Extracted values ranged from 0.43 to 0.57, indicating acceptable convergent validity for most factors. Although Factor 1's Average Variance Extracted was slightly below the commonly recommended threshold of 0.50, the high Composite Reliability suggests that the construct's reliability remains adequate. See Table 2 for factor loadings and validity statistics.

**TABLE 2:** Factor loadings of the confirmatory factor analysis (CFA).

Items	Short names	Factor 1	Factor 2	Factor 3	*R* ^2^
TR3	Impulsivity	0.60	—	—	0.36
**TR5**	**Racing thoughts**	**0.59**	—	—	**0.35**
TR7	Low mood	0.66	—	—	0.44
**TR12**	**Disorganized thinking**	**0.32**	—	—	**0.10**
TR15	Emptiness	0.67	—	—	0.45
TR19	Mood cycle	0.71	—	—	0.50
TR21	Irritability	0.71	—	—	0.50
TR23	Mood lability	0.68	—	—	0.48
TR24	Disconnected thoughts	0.74	—	—	0.55
TR26	Identity confusion	0.70	—	—	0.49
TR27	Poverty of speech	0.61	—	—	0.37
TR34	Emotional numbness	0.72	—	—	0.52
TR37	Confused thinking	0.75	—	—	0.56
TR6	Auditory hallucinations	—	0.71	—	0.50
TR9	Sound distraction	—	0.74	—	0.55
TR17	Reality confusion	—	0.79	—	0.62
TR18	Precepted oddity in familiar surroundings	—	0.75	—	0.56
TR29	Pseudo hallucinations	—	0.74	—	0.74
TR30	Suspiciousness	—	0.80	—	0.64
TR33	Paranoia	—	0.67	—	0.45
TR13	Pressured speech/talkative	—		0.73	0.53
TR14	Elevated self‐confidence	—		0.79	0.62
TR16	Elevated mood or energy	—		0.77	0.59
TR20	Increased goal directed activities	—		0.72	0.52
TR22	Grandiosity	—		0.81	0.65
TR25	Idea of reference	—		0.72	0.52
Alpha		0.91	0.89	0.88	
Omega		0.88	0.84	0.84	
AVE		0.43	0.55	0.57	

*Note:* Factor loadings represent standardized estimates from the CFA. *R* ^2^ indicates the variance explained by each factor. Given the ordinal and skewed nature of the data, Omega and AVE were prioritised for evaluating reliability and validity, while Alpha was reported for reference. Items with loadings below 0.60 are bolded.

Abbreviations: Alpha = ordinal Cronbach's alpha; AVE = average variance extracted; Omega = ordinal McDonald's omega.

**FIGURE 2:**
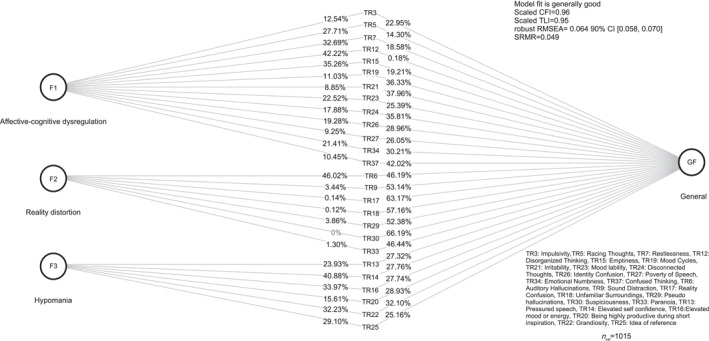
Bi‐factor analysis model with standardized factor loadings squared. The percentage associated with each item represent the standardized factor loadings squared, which indicates the proportion of variance in each item explained by that factor.

To further evaluate the factor structure, we tested a bi‐factor model. This model posits one general factor capturing shared variance across all items, alongside three independent factors. The bi‐factor model demonstrated an improved fit relative to the three‐factor model (Comparative fit index = 0.96, Tucker Lewis Index = 0.95, Root Mean Square Error of Approximation = 0.064 [CI 0.058, 0.070], Standardised Root Mean Square Residual = 0.049). The model can be best described as an incomplete bi‐factor model, whereby some items only load onto the general factor and not on any specific factor. See Table 3 for factor loadings and Figure 2 for a representation of this analysis. The bifactor structure demonstrated strong saturation of the general factor, with an Explained Common Variance of 64% and a Percent of Uncontaminated Correlations of 65%. The total omega (*ω*) was 0.93, and the omega hierarchical (*ω*_*h*_) was 0.75, indicating good overall reliability. At the factor level, construct replicability was acceptable for Factor 1 (*H* = 0.79) and Factor 3 (*H* = 0.72), whereas Factor 2 demonstrated weaker replicability (*H* = 0.49). This suggests that Factor 2, Reality Distortion, may not represent a distinct or reliably measured construct within this model. Consequently, interpretations involving this specific factor should be made with caution.

**TABLE 3:** Factor loadings of the Bi‐factor model.

Items	Short names	GF	F1	F2	F3	*R* ^2^ (GF/SF)
TR3	Impulsivity	0.48	0.35			0.23/0.13
**TR5**	**Racing thoughts**	**0.38**	**0.53**			**0.14/0.28**
**TR7**	**Low mood**	**0.43**	**0.57**			**0.19/0.33**
**TR12**	**Disorganized thinking**	**0.04**	**0.65**			**0.002/0.42**
**TR15**	**Emptiness**	**0.44**	**0.59**			**0.19/0.35**
TR19	Mood cycle	0.60	0.33			0.36/0.11
TR21	Irritability	0.62	0.30			0.38/0.09
TR23	Mood lability	0.50	0.48			0.25/0.23
TR24	Disconnected thoughts	0.60	0.42			0.36/0.18
TR26	Identity confusion	0.54	0.44			0.29/0.19
TR27	Poverty of speech	0.51	0.30			0.26/0.09
TR34	Emotional numbness	0.55	0.46			0.30/0.21
TR37	Confused thinking	0.65	0.32			0.42/0.10
TR6	Auditory hallucinations	0.68		0.68		0.46/0.46
TR9	Sound distraction	0.73		0.19		0.53/0.03
TR17	Reality confusion	0.80		0.04		0.63/0.002
TR18	Precepted oddity in familiar surroundings	0.76		0.04		0.57/0.002
TR29	Pseudo hallucinations	0.72		0.20		0.52/0.04
TR30	Suspiciousness	0.81		−0.01		0.66/0.0001
TR33	Paranoia	0.68		−0.11		0.46/0.01
TR13	Pressured speech/talkative	0.52			0.49	0.27/0.24
**TR14**	**Elevated self‐confidence**	**0.53**			**0.64**	**0.28/0.41**
**TR16**	**Elevated mood or energy**	**0.53**			**0.58**	**0.28/0.34**
TR20	Increased goal directed activities	0.54			0.40	0.29/0.16
TR22	Grandiosity	0.57			0.57	0.32/0.32
**TR25**	**Idea of reference**	**0.50**			**0.54**	**0.25/0.29**

*Note:* Items are bolded when their specific factor loadings exceed their general factor loadings.

Abbreviations: F1–F3 = Factor 1–Factor 3; GF = General factor; GF/SF = proportion of variance explained by general or specific factors.

These analyses used the refined TRACE‐26 measure. To assess whether TRACE correlated with relevant psychometric and personality variables, we correlated factor scores and total TRACE scores with these measures. These results are summarised in Table 4. There are strong correlations between sub‐factors and related constructs, for example, between Trace Reality Distortion and the PQ16, *r* (2,035) = 0.81, *p* < 0.001, and between Trace Dysregulation and neuroticism, *r* (2,035) = 0.44, *p* < 0.001. Importantly, an early‐risk screener should identify relevant experiences that are not diagnostic of any particular disorder and TRACE correlates well will measures such as loneliness, *r* (2,035) = 0.43, *p* < 0.001, and hopelessness, *r* (2,035) = 0.32, *p* < 0.001.

**TABLE 4:** Pearson correlations of TRACE dimensions and total sum scores with relevant psychometrics.

	PQ16	PHQ8	GAD7	WEMWS	Espie	BF Ext.
TR dys	0.70***	0.67***	0.69***	−0.39***	−0.51***	−0.18***
TR psy	0.81***	0.47***	0.47***	−0.10***	−0.31***	−0.05*
TR man	0.57***	0.20***	0.22***	0.21***	−0.07**	0.06**
TR total	0.83***	0.60***	0.62***	−0.21***	−0.42***	−0.11***

	**BF Ag.**	**BF Con.**	**BF Neu.**	**BF Op.**	**Loneliness**	**Hopelessness**
TR dys	−0.15***	−0.19***	0.44***	0.15***	0.50***	0.39***
TR psy	−0.13***	−0.11***	0.16***	0.05*	0.32***	0.24***
TR man	0.02	0.03	−0.09***	−0.01	0.08***	0.05*
TR total	−0.12***	−0.14***	0.29***	0.10***	0.43***	0.32***

	**Religiosity**	**HoSP**	**Neg. self**	**Pos. self**	**Neg. other**	**Pos. other**
TR dys	0.07**	0.47***	0.52***	−0.27***	0.40***	−0.20***
TR psy	0.23***	0.43***	0.35***	−0.03	0.36***	−0.06**
R Man	0.35***	0.24***	0.08***	0.27***	0.21***	0.17***
TR total	0.21***	0.48***	0.45***	−0.10***	0.41***	−0.09***

Abbreviations: BF Agg: Big Five Agreeableness; BF Con: Big Five Conscientiousness; BF Ext: Big Five Extraversion; BF Neu: Big Five Neuroticism; BF Op: Big Five Openness; Espie: Espie Sleep Indicator; GAD7: General Anxiety Disorder‐7; HoSP: History of Social Punishment Scale; Neg Other: Brief Core Schema Scale Negative Other; Brief Core Schema Scale Positive Other; Neg. self: Brief Core Schema Scale Negative Self; PHQ8: Patient Health Questionnaire‐8; Pos self: Brief Core Schema Scale Positive self; PQ16: Prodromal questionnaire 16 items; TR dys: TRACE Affective‐cognitive dysregulation; TR man: TRACE mania; TR psy: TRACE psychosis; WEMWS: The Warwick‐Edinburgh Mental Wellbeing Scales.

* *p* < 0.05.

** *p* < 0.01.

*** *p* < 0.001.

### 3.6 Determining the High‐Risk Cut‐Off—Combined Dataset

A high‐risk group was defined as the top decile of TRACE scores (≥ 41). Elevated risk was defined as ≥ 6 positively endorsed items on the PQ16 and low‐risk was categorised as < 6 positively‐endorsed items on the PQ16.

A receiver operating characteristic curve was conducted to determine an optimal cutoff value to discriminate between moderate and low‐risk individuals, using PQ16 groups as the comparison variable. The analysis revealed that TRACE scores were a significant predictor of PQ16 scores, with an area under the curve of 0.872, 95% CI [0.858, 0.887], indicating good discriminative ability. The optimal cut‐off for identifying elevated risk, as indicated by Youden's index (Sensitivity + Specificity—1), is a Total Trace distress score of 19.5 (rounded to 19), which maximizes sensitivity (80%) with acceptable specificity (23%). Although specificity is low, prioritising sensitivity is preferable for a first‐stage risk screener.

Independent ANOVAs between high‐risk (TRACE ≥ 41; *n* = 206), moderate risk (TRACE 19—40; *n* = 799) and low‐risk (TRACE < 19; *n* = 1032) individuals reveal significant differences between TRACE‐risk groups across all measured variables (See Table 5 for descriptive statistics and Table 6 for a summary of ANOVA statistics).

**TABLE 5:** Means and standard deviations for each outcome variable by risk group.

Outcome variable	Low risk (*n* = 1032)		Moderate risk (*n* = 799)		High risk (*n* = 206)	
	*M*	SD	*M*	SD	*M*	SD
PQ16	2.94	3.17	10.98	6.43	24.42	8.79
PHQ8	6.20	4.94	11.32	5.49	15.64	5.66
GAD7	4.78	4.57	10.07	5.40	14.12	5.41
WEMWS	45.68	10.86	42.08	11.34	39.95	14.04
Espie	21.42	7.33	16.83	7.36	13.02	7.84
Neuroticism	2.97	0.98	3.35	0.98	3.66	0.93
Loneliness	5.21	1.78	6.44	1.77	7.11	1.67
Hopelessness	3.51	2.25	4.43	2.29	5.47	2.39
HoSP	24.11	10.76	18.42	9.59	11.65	8.78
Negative self	1.77	3.52	4.68	5.76	8.54	7.80
Positive self	12.31	7.14	11.30	7.85	10.89	8.60
Negative other	3.68	4.85	6.62	6.41	11.19	7.70
Positive other	9.14	6.48	8.16	6.63	8.06	7.87

*Note:* Brief Core Schema Scale subscales: negative self, positive self, negative other, positive other.

Abbreviations: Espie: Espie Sleep Indicator; GAD7: General Anxiety Disorder‐7; Hopelessness: 2‐Item hopelessness Scale; HoSP: History of Social Punishments 11 items; loneliness: 3‐item loneliness scale; Neuroticism: Big Five Neuroticism subscale; PHQ8: Patient Health Questionnaire‐8; PQ16: Prodromal questionnaire 16 items; WEMWS: The Warwick‐Edinburgh Mental Wellbeing Scales.

**TABLE 6:** Summary of ANOVA results for outcome variables.

Outcome variable	*F* (22,034)	*p*	Partial *η* ^2^
PQ16	1528.78	< 0.001	0.601
PHQ	390.81	< 0.001	0.278
GAD7	435.19	< 0.001	0.300
WEMWS	34.76	< 0.001	0.033
EPSIE	156.12	< 0.001	0.133
Neuroticism	61.40	< 0.001	0.057
Loneliness	163.58	< 0.001	0.139
Hopelessness	79.08	< 0.001	0.072
HoSP	214.92	< 0.001	0.174
Negative self	184.99	< 0.001	0.154
Positive self	5.51	0.004	0.005
Negative other	162.90	< 0.001	0.138
Positive other	5.75	0.003	0.006

*Note:* Brief Core Schema Scale subscales: negative self, positive self, negative other, positive other.

Abbreviations: Espie: Espie Sleep Indicator; GAD7: General Anxiety Disorder‐7; Hopelessness: 2‐Item hopelessness Scale; HoSP: History of Social Punishments 11 items; loneliness: 3‐item loneliness scale; Neuroticism: Big Five Neuroticism subscale; PHQ8: Patient Health Questionnaire‐8; PQ16: Prodromal questionnaire 16 items; WEMWS: The Warwick‐Edinburgh Mental Wellbeing Scales.

For every significant ANOVA, we conducted post‐hoc *t*‐tests. Exact *p*‐values are reported, but to control familywise error, we use an adjusted alpha level of 0.017 to infer significant differences between the subgroups. See Table 7 for a summary of these results. Unsurprisingly, the biggest differences were typically between the low‐ and the high‐risk groups. The high‐TRACE group had significantly higher levels of early‐stage psychosis, higher depression, lower wellbeing, worse sleep and felt more lonely and more hopeless than the low‐TRACE group—collectively indicating good concurrent validity of the TRACE measure. Additionally, they endorsed more negative statements about others and fewer about themselves. They also had more neuroticism, felt lonelier and more hopeless and had a greater history of social punishments.

**TABLE 7:** Summary of *t*‐tests for outcome variables.

Outcome variable	High versus Moderate *t* (1003)	Cohen's *d*	*p*	High versus Low *t* (1236)	Cohen's *d*	*p*	Moderate versus. Low *t* (1829)	Cohen's *d*	*p*
PQ16	24.64	1.93	< 0.001	61.18	4.67	< 0.001	35.08	1.65	< 0.001
PHQ8	9.99	0.78	< 0.001	24.41	1.86	< 0.001	20.97	0.99	< 0.001
GAD7	9.59	0.75	< 0.001	25.93	1.98	< 0.001	22.67	1.07	< 0.001
WEMWS	−2.28	−0.18	0.023	−6.56	−0.50	< 0.001	−6.90	−0.33	< 0.001
Espie	−6.54	−0.51	< 0.001	−14.85	−1.13	< 0.001	−13.27	−0.63	< 0.001
Neuroticism	4.10	0.32	< 0.001	9.35	0.71	< 0.001	8.26	0.39	< 0.001
Loneliness	4.91	0.38	< 0.001	14.08	1.07	< 0.001	14.65	0.69	< 0.001
Hopelessness	5.77	0.45	< 0.001	11.26	0.86	< 0.001	8.53	0.40	< 0.001
Negative self	7.93	0.62	< 0.001	19.61	1.50	< 0.001	13.32	0.63	< 0.001
Positive self	−0.65	−0.05	0.517	−2.50	−0.19	0.012	−2.87	−0.14	0.004
Negative other	8.73	0.87	< 0.001	18.13	1.38	< 0.001	11.19	0.53	< 0.001
Positive other	−0.18	−0.18	0.861	−2.10	−0.16	0.036	−3.19	−0.15	0.001
HoSP	7.40	0.58	< 0.001	17.87	1.36	< 0.001	15.70	0.74	< 0.001

*Note:* Brief Core Schema Scale subscales: negative self, positive self, negative other, positive other.

Abbreviations: Espie: Espie Sleep Indicator; GAD7: General Anxiety Disorder‐7; Hopelessness: 2‐Item hopelessness Scale; HoSP: History of Social Punishments 11 items; loneliness: 3‐item loneliness scale; Neuroticism: Big Five Neuroticism subscale; PHQ8: Patient Health Questionnaire‐8; PQ16: Prodromal questionnaire 16 items; WEMWS: The Warwick‐Edinburgh Mental Wellbeing Scales.

Low versus moderate risk group typically exerted bigger differences than the high‐moderate comparisons, supporting the notion that the moderate risk group is at elevated risk for mental health problems.

### 3.7 Incremental Validity—Combined Dataset

To determine whether TRACE explains additional variance beyond existing prodromal measures, we tested whether it explains additional variance in wellbeing scores beyond that of the PQ16, which measures prodromal psychosis. We ran a hierarchical regression, controlling for age and sex in block one, adding PQ16 to block two, and TRACE to block three. In the first model, age and sex accounted for a modest 5.2% of the variance in wellbeing (Adjusted *R* ^2^ = 0.051, *F* (2,2034) = 55.56, *p* < 0.001). Adding PQ16 in the second model increased the variance explained to 8.0% (Adj. *R* ^2^ = 0.079, *F* (3,2033) = 59.24, *p* < 0.001). In the final model, where TRACE was added, the explained variance further increased to 9.9% (Adjusted *R* ^2^ = 0.097, *F* (4,2032) *=* 55.76, *p* < 0.001). Furthermore, while PQ16 showed a significant negative relationship with wellbeing in the second model (*β* = −0.169, *p* < 0.001), its effect became non‐significant in the presence of TRACE (*β* = 0.035, *p* = 0.361). Whereas TRACE scores demonstrated a significant negative relationship with wellbeing in the third model (*β* = −0.245, *p* < 0.001). These results suggest that the TRACE provides incremental validity over the PQ16 in predicting wellbeing/distress.

## 4 Discussion

The current study contributes to a growing body of research on early detection of an at‐risk mental state by developing an easy‐to‐use, pluripotent, self‐report measure, suitable for both adolescents and young adults. The TRACE scale has a reliable structure, good incremental validity and a pre‐specified cutoff which predicts higher levels of at‐risk psychosis, depression, lower well‐being, worse sleep quality, and higher loneliness. These findings provide concurrent validity for the TRACE scale, linking it to established mental health indicators and support its use as a risk indicator. Its short self‐report nature means it is easy to use in a wide range of contexts beyond those help‐seeking for mental health issues. For example, people with physical health problems or those who are experiencing interpersonal difficulties.

The TRACE shows promise for identifying both a high‐risk group and a moderate‐risk group. In this study, the moderate‐risk group comprised 39% of the sample. Although this percentage seem high, it reflects the rising mental‐health concerns among young people. According to official NHS statistics, 23% of 17–19‐year‐olds, and 22% of 20‐ to 25‐year‐olds have a probable mental disorder (NHS digital, 2023). Early‐stage screening should focus on identifying as many at‐risk individuals as possible, even if it means that some low‐risk individuals may also be identified. Equally, there were significant differences between all three risk groups and the TRACE was able to identify a group who is at high‐risk of associated mental‐health difficulties.

We composed our measure based on early indicators of mania/bipolar disorder, psychosis and borderline personality. The factor analysis confirms that our questions cluster in meaningful ways around these disorders. However, the incomplete bi‐factor analysis suggests that we should try to collapse these factors together to conceptualise a broad pluripotent at‐risk state, which accounts for general psychopathology.

This finding is consistent with the HiTOP model's framework (Kotov et al. 2017), which emphasizes the co‐occurrence and dimensional nature of psychopathology. In this context, the incomplete bi‐factor solution can be interpreted as evidence of both disorder‐specific patterns and a shared underlying vulnerability that cuts across traditional diagnostic boundaries. The presence of a general factor suggests that symptoms may emerge from a pluripotent at‐risk state, supporting the CHARMS framework (Hartmann et al. 2021).

Additionally, beyond identifying shared variance across disorders, TRACE captures the co‐occurrence and interplay of early symptoms. This allows researchers to investigate network‐based models of psychopathology (Borsboom 2017; Robinaugh et al. 2020), which postulate that symptoms interact to form networks that may spiral into disorder, rather than assuming a latent disorder causes all symptoms. When investigating the at‐risk mental state, it is important to see whether certain clusters of symptoms become strongly connected, creating a vulnerable or self‐perpetuating system, so as to better understand the time course and development of mental health issues.

This is the first step in developing and verifying the TRACE measure. We plan to further develop and validate this tool in both longitudinal research projects and clinical settings. We are currently running a prospective cohort study, where we are collecting self‐report data from an undergraduate population and will assess the extent to which TRACE scores predict self‐report measures of severe mental health issues, including bipolar disorder, borderline personality and psychosis. In this study we will be able to validate the recommended TRACE cut‐offs using longitudinal data. We will monitor how symptoms change over time and how this impacts TRACE scores. We will also investigate whether TRACE can successfully differentiate between certain developmental conditions and mental illness. Some features identified by the TRACE, such as social withdrawal, may also occur in conditions such as autism. In this follow‐up study we measure autistic traits and collect data on neurodivergent conditions, so we can investigate whether TRACE can distinguish emerging psychopathology from stable developmental traits. In a further follow‐up study, we plan to validate the TRACE against a long‐form clinical interview, such as the CAARMS. Further validation will come from measuring TRACE scores in a help‐seeking, clinical population. This research will verify if help‐seeking individuals, who are in distress, score higher on TRACE than general population participants.

In summary, the newly developed TRACE measure is designed to screen for several severe mental health conditions in adolescents and young adults in a single measure. This research significantly advances the early detection of the at‐risk mental state. It offers practical benefits for pre‐screening, as well as theoretical insights into the structure of the pluripotent at‐risk state.

## Author Contributions

**Jayne Pickering:** investigation, writing – original draft, methodology, formal analysis, project administration, data curation. **Wen Shao:** conceptualization, methodology, writing – review and editing, formal analysis, software, visualization. **Scott Weich:** methodology, writing – review and editing. **Caroline F. Dalton:** methodology, writing – review and editing. **Myles Jones:** methodology, writing – review and editing. **Chris D. J. Taylor:** methodology, writing – review and editing. **Markus Reuber:** methodology, writing – review and editing. **Jessica Kingston:** methodology, writing – review and editing. **Chris Gibbs:** methodology, writing – review and editing. **Richard P. Bentall:** conceptualization, methodology, supervision, writing – review and editing.

## Conflicts of Interest

The authors declare no conflicts of interest.

## Supporting information


Supporting Information S1

## Data Availability

The data that support the findings of this study are openly available in Open Science Framework at https://osf.io/2spwf/.

## Endnotes

^1^
We also included a religiosity measure, as part of a separate research project.

^2^
See supplementary materials for software and further methodological details.
