# Supplementary material for: Developing Trace: A Transdiagnostic Screening Tool for Early Detection of a Pluripotent at‐Risk Mental State
Source: Int J Methods Psychiatr Res. 2025 Dec 3;34(4):e70046. doi: 10.1002/mpr.70046 (PMC12675136; doi:10.1002/mpr.70046)
Supplement: Supplementary file 1 — Supporting Information S1 [file MPR-34-e70046-s001.pdf]

Supplementary materials

1. ***Piloted 37-item version of TRACE***

Instructions: Please indicate whether you have had the following thoughts, feelings and experiences in the **past month** by selecting "true" or "false" for each item. Do not include experiences that occurred only while under the influence of alcohol, drugs or medications that were not prescribed to you. If you experience an item, we will ask you to indicate how distressing that experience was for you.

Questionnaire format:

[Statement] True/false.

[If statement endorsed] How much distress did you experience?

Original 37 items (items in bold were removed from the final 26-item version).

TR1. **I have had motivation to do things (R)**

TR2. **There have been times when I was so restless that it was impossible for me to sit still**

TR3. I have behaved impulsively and later regretted it

TR4. **I have been interested in getting to know other people (R)**

TR5. I have often found that my thoughts were racing

TR6. I have heard sounds that were not there

TR7. During the last month, my mood became extremely low for a period of a few days

TR8. **Somebody who I thought was going to be very special quickly turned out to be a huge disappointment**

TR9. I have felt suddenly distracted by distant sounds that I am not normally aware of

TR10. **I have been concerned that my closest friends and co-workers were not really loyal or trustworthy**

TR11. **There have often been times when I had such an excess of energy that I felt little need to sleep at night**

TR12. I have found it easy to organise my thoughts or to find the right words (R)

TR13. I often got into excited moods where it was almost impossible for me to stop talking

TR14. I often had moods where I felt so energetic and optimistic that I felt I could outperform almost anyone at anything

TR15. I have often felt empty

TR16. I have had periods of extreme happiness and high energy lasting several days or more

TR17. I have confused whether something I experienced was real or imaginary

TR18. My familiar surroundings have occasionally appeared to be changed, unreal, or strange

TR19. I have had periods lasting several days or more when I felt depressed or irritable, and then other periods of several days or more when I felt extremely high, elated, and overflowing with energy

TR20. I did most of my work during brief periods of intense inspiration

TR21. There have been times when, although I was feeling unusually happy and intensely energetic, almost everything got on my nerves and made me irritable or angry (other than related to the menstrual cycle)

TR22. I have thought that I am very important or have abilities that are out of the ordinary

TR23. When something bad happened, my mood changed very quickly and it took me a long time to calm down

TR24. I have sometimes had too many thoughts going through my head without any connection between them

TR25. I have felt that everyday things had a special meaning just for me

TR26. I have often felt that I have no idea of who I am or that I have no identity

TR27. I have had nothing to say or very little to say

TR28. **I have got so angry, I have felt like I might lose control**

TR29. I have mistaken shadows for people or noises for voices

TR30. I have often had a feeling that something strange and unusual is happening around me

TR31. **I have made desperate efforts to avoid feeling abandoned or being abandoned (e.g., repeatedly called someone to reassure myself that he or she still cared, begged them not to leave me, clung to them physically)**

TR32. **In the last month, I have engaged in very pleasurable but reckless and risky activities**

TR33. I have felt that people were plotting against me or planning to harm me

TR34. I have felt emotionally numb

TR35. **I have been preoccupied with unusual, magical, or supernatural things or ideas**

TR36. **I have had thoughts that other people would think are strange**

TR37. My thinking has felt confused, muddled, or disturbed in some way

Scoring: None (0), Mild (1), Moderate (2), Severe (3), then summed. Total scores range from 0 to 78.

2. ***Methodology step three: preliminary list of 214 questions (including 3 duplicates, marked by*) and 79 symptom/trait criteria.***

Model	Sub-dimension	Symptom/trait	Questions	No.	Questionnaire
Negative	Emotional expression	Poor rapport	I am poor at returning social courtesies and gestures	1	BSQSP and CPRQ
Negative	Emotional expression	Poor rapport	Increased problems with relationships (partner, family, work)	2	Self-screen Prodrome
Negative	Emotional expression	Poor rapport	I tend to keep my feelings to myself	3	BSQSP, PS-R, and CPRQ
Negative	Emotional expression	Poor rapport	Is it hard to establish a connection or do you feel at a distance when you are talking with others?	4	YPARQ-B
Negative	Emotional expression	Emotions less recognisable and appropriate	I do not have an expressive and lively way of speaking	5	BSQSP and CPRQ
Negative	Emotional expression	Inappropriate affect	People have said that I seemed "spacey" or "out of it"	6	PQ
Negative	Emotional expression	Inappropriate affect	People have found me to be aloof and distant	7	PQ
Negative	Emotional expression	Diminished emotional responsiveness	Do you ever feel that you are not a very animated person?	8	CAPE
Negative	Emotional expression	Diminished emotional responsiveness	I have had difficulty expressing my feelings as well as I used to	9	PQ

Negative	Emotional expression	Lack of spontaneity and flow of conversation	Do you ever feel that you are lacking in spontaneity?	10	CAPE
Negative	Emotional expression	Lack of spontaneity and flow of conversation	I have had nothing to say or very little to say	11	PQ
Negative	Emotional expression	Lack of spontaneity and flow of conversation	Do you ever feel that you are not much of a talker when you are conversing with other people?	12	CAPE
Negative	Social anhedonia	Does not initiate contact	Do you tend to avoid social activities with others?	13	YPARQ-B
Negative	Social anhedonia	Does not initiate contact	I have had little interest in getting to know other people	14	PQ (Prodromal Questionnaire)
Negative	Social anhedonia	Does not initiate contact	I have avoided social activities with other people	15	PQ (Prodromal Questionnaire)
Negative	Social anhedonia	Tends to recede into the background	I am mostly quiet when with others	16	BSQSP and CPRQ
Negative	Social anhedonia	Lack of close friends	I feel I cannot get close to people	17	BSQSP and CPRQ
Negative	Avolition	Impairment in goal directed activities	Do you ever feel that you can never get things done?	18	CAPE
Negative	Avolition	Impairment in goal directed activities	I have been less able to do usual activities or tasks	19	PQ
Negative	Avolition	Impairment in goal directed activities	Difficulties in carrying out ordinary routine activities for the last 1 week	20	PROD-screen
Negative	Avolition	Low drive, energy, or productivity	Have you felt like your thinking, speaking or movements have been slowing down noticeably? Has your empathy with others changed?	21	ERIraros
Negative	Avolition	Low drive, energy, or productivity	I have felt unable to carry out everyday tasks because of fatigue or lack of motivation	22	PQ
Negative	Avolition	Low drive, energy, or productivity	I have been less interested in school or work	23	PQ
Negative	Avolition	Low drive, energy, or productivity	Do you ever feel that you are lacking in motivation to do things?	24	CAPE
Negative	Avolition	Low drive, energy, or productivity	Do you ever feel that you are lacking in energy?	25	CAPE
Negative	Avolition	Low drive, energy, or productivity	Do you ever feel that you are spending all your days doing nothing?	26	CAPE

Negative	Avolition	Low drive, energy, or productivity	Lack of energy, drive, initiative or interest	27	Self-screen Prodrome and EDPCC
Negative	Experience of emotions and self	Emotions disappearing	Do you ever feel that your emotions are blunted?	28	CAPE
Negative	Experience of emotions and self	Emotions disappearing	Do you ever feel that your feelings are lacking in intensity?	29	CAPE
Negative	Experience of emotions and self	Feeling depersonalised at a distance from self	I have felt like I was looking at myself as in a movie, or that I was a spectator in my own life	30	PQ
Negative	Experience of emotions and self	Feeling depersonalised at a distance from self	Have you felt at a distance from yourself, as if you were outside your own body?	31	YPARQ-B
Negative	Experience of emotions and self	Feeling profoundly changed unreal or strange	When I looked at a person, or myself in a mirror, I have seen the face change right before my eyes.	32	PQ
Negative	Experience of emotions and self	Feeling profoundly changed unreal or strange	Have your familiar surroundings occasionally appeared to be changed, unreal or strange? Or have you ever had the feeling that you were not yourself or that you were unreal or strange to yourself, e.g. When looking in the mirror?	33	ERIraros
Negative	Experience of emotions and self	Loss of sense of self	Do you ever feel that your mind is empty?	34	CAPE
Negative	Experience of emotions and self	Sense of having no feelings	Do you ever feel that you experience few or no emotions at important events?	35	CAPE
Negative	Ideational richness	Unable to make sense of familiar phrases	Do you sometimes feel that you cannot immediately understand spoken or written words, even though these are familiar to you?	36	PCA
Negative	Ideational richness	Unable to make sense of familiar phrases	Do you sometimes catch bits and pieces of conversation that you are then unable to link intelligibly?	37	PCA
Negative	Ideational richness	Unable to make sense of familiar phrases	I often have to reflect over the meaning of very common words.	38	YSR and ESI
Negative	Ideational richness	Unable to make sense of familiar phrases	If someone speaks with long sentences, I have difficulties to grasp the meaning correctly.	39	ESI and EDPCC
Negative	Ideational richness	Unable to make sense of familiar phrases	If someone speaks to me, I often have trouble grasping	40	ESI

			the meaning of the words correctly.		
Negative	Ideational richness	Unable to make sense of familiar phrases	Common words sometimes seem to have a peculiar strange meaning.	41	ESI
Negative	Ideational richness	Difficulty performing role functions	I have been in danger of failing out of school, or of being fired from my job	42	PQ
Negative	Ideational richness	Difficulty performing role functions	Marked decline in performance, possibly with difficulties at work or school	43	Self-screen Prodrome
Negative	Ideational richness	Difficulty performing role functions	Have you become significantly less persistent, motivated or productive at work, school, in your studies, occupational training, search for work? Has your performance deteriorated?	44	ERIraros
Negative	Ideational richness	Difficulty in productive relationships at work or school	I have not gotten along well with people at school or at work	45	PQ
Disorganisation	Impairment in personal hygiene	Self neglect	I have noticed that I am less interested than I used to be in keeping clean or dressing well	46	PQ
Disorganisation	Impairment in personal hygiene	Self neglect	Do you ever feel that you are neglecting your appearance or personal hygiene?	47	CAPE
Disorganisation	Bizarre thinking	Strange, fantastic or bizarre ideas	Feelings, thoughts or behaviours that could be considered weird or peculiar	48	PROD-screen
Disorganisation	Bizarre thinking	Strange, fantastic or bizarre ideas	People have said that my ideas were strange or illogical	49	PQ
Disorganisation	Bizarre thinking	Strange, fantastic or bizarre ideas	My thinking has felt confused, muddled, or disturbed in some way*	50	PQ
Disorganisation	Bizarre thinking	Strange, fantastic or bizarre ideas	Do you sometimes have too many thoughts going through your head without any connection between them?	51	PCA
Disorganisation	Bizarre thinking	Strange, fantastic or bizarre ideas	I have thoughts that other people would think are strange	52	YSR
Disorganisation	Trouble with focus and attention	Trouble with short term memory	I have had trouble remembering things	53	PQ
Disorganisation	Trouble with focus and attention	Difficulty in focusing to new stimuli	When I watch television, it is difficult for me to follow the pictures and words and to catch the story simultaneously	54	ESI

Disorganisation	Trouble with focus and attention	Difficulty concentrating	I cannot focus on a task and need to take frequent breaks while working (studying).	55	BSQSP and CPRQ
Disorganisation	Trouble with focus and attention	Difficulty concentrating	I feel mentally insufficient and easily fatigued while thinking or reading	56	BSQSP and CPRQ
Disorganisation	Trouble with focus and attention	Difficulty concentrating	I have had difficulty concentrating, listening or reading	57	PQ
Disorganisation	Trouble with focus and attention	Failure in focused alertness	I have had trouble focusing on one thought at a time	58	PQ
Disorganisation	Trouble with focus and attention	Failure in focused alertness	I have felt suddenly distracted by distant sounds that I am not normally aware of	59	PQ
Disorganisation	Trouble with focus and attention	Failure in focused alertness	I have been distracted by noises or other people talking	60	PQ
Disorganisation	Trouble with focus and attention	Failure in focused alertness	Do you find yourself constantly thinking about experiences or conversations that are, in fact, irrelevant, when you would rather be focusing on other matters?	61	PCA
Disorganisation	Odd behaviour and appearance	Odd behaviour	Plays with own sex parts in public	62	CBCL
Disorganisation	Odd behaviour and appearance	Odd behaviour	I do things other people think are strange	63	YSR
Disorganisation	Odd behaviour and appearance	Odd behaviour	Have you pursued interests or behaviours that other people regarded as odd?	64	ERIraros
Disorganisation	Odd behaviour and appearance	Odd behaviour	Repeats certain acts over and over; compulsions	65	EDPCC
Disorganisation	Odd behaviour and appearance	Odd behaviour	I have engaged in some eccentric (odd) habits	66	PQ
Disorganisation	Odd behaviour and appearance	Odd behaviour	People have commented on my unusual mannerisms and habits	67	PQ
Disorganisation	Odd behaviour and appearance	Odd behaviour	I have thought that I am an odd, unusual person	68	PQ
Disorganisation	Odd behaviour and appearance	Preoccupied with and/or interactive with own thoughts	I have been talking to myself	69	PQ

Disorganisation	Odd behaviour and appearance	Preoccupied with and/or interactive with own thoughts	Changes in behaviour (e.g. Loud soliloquy in public)	70	Self-screen Prodrome
Disorganisation	Odd behaviour and appearance	Preoccupied with and/or interactive with own thoughts	Experience of thoughts running wild or difficulty in controlling the speed of thoughts	71	PROD-screen
General	Dysphoric mood	Anxiety, panic, multiple fears and phobias	I feel nervous when giving a speech in front of a large group of people	72	BSQSP and CPRQ
General	Dysphoric mood	Anxiety, panic, multiple fears and phobias	I cannot deal with the pressures associated with crowds	73	BSQSP and CPRQ
General	Dysphoric mood	Anxiety, panic, multiple fears and phobias	I can't get my mind [away from] certain thoughts	74	YSR
General	Dysphoric mood	Diminished interest in pleasurable activities	Do you ever feel that you have only few hobbies or interests?	75	CAPE
General	Dysphoric mood	Diminished interest in pleasurable activities	I have felt unable to enjoy things that I used to enjoy	76	PQ
General	Dysphoric mood	Diminished interest in pleasurable activities	I have felt uninterested in the things I used to enjoy	77	PQ
General	Dysphoric mood	Feelings of loss of energy	I feel lethargic whatever I do	78	BSQSP and CPRQ
General	Dysphoric mood	Feelings of worthlessness and/or guilt	I always mess up whatever I do	79	BSQSP and CPRQ
General	Dysphoric mood	Feelings of worthlessness and/or guilt	I have felt very guilty	80	PQ
General	Dysphoric mood	Feelings of worthlessness and/or guilt	I have felt worthless	81	PQ
General	Dysphoric mood	Feelings of worthlessness and/or guilt	Do you ever feel like a failure?	82	CAPE
General	Dysphoric mood	Irritability, hostility, rage	I have felt angry, easily irritated or offended	83	PQ
General	Dysphoric mood	Irritability, hostility, rage	Over-sensitivity, more easily hurt or upset	84	Self-screen Prodrome and YSR
General	Dysphoric mood	Poor or increased appetite	Poor appetite	85	EDPCC
General	Dysphoric mood	Restless, agitation, tension	I have felt more nervous or anxious, and have found it hard to relax	86	PQ

General	Dysphoric mood	Restless, agitation, tension	Nervousness, feeling tense	87	Self-screen Prodrome
General	Dysphoric mood	Restless, agitation, tension	Have you often felt nervous, tense or restless? Has this led to arguments with others even about minor issues? Have you been more active than usual? Maybe so active that others might have thought that there was something wrong with you?	88	ERIraros
General	Dysphoric mood	Suicidal thoughts	Do you ever feel as if you do not want to live anymore?	89	CAPE
General	Dysphoric mood	Suicidal thoughts	Deliberately harms self or attempts suicide	90	CBCL
General	Dysphoric mood	Suicidal thoughts	I deliberately try to hurt or kill myself	91	YSR
General	Dysphoric mood	Unstable mood	My moods have been highly changeable and unstable	92	PQ
General	Dysphoric mood	Unstable mood	I have felt unhappy or depressed	93	PQ
General	Dysphoric mood	Unstable mood	Do you ever feel pessimistic about everything?	94	CAPE
General	Dysphoric mood	Unstable mood	Do you ever feel as if there is no future for you?	95	CAPE
General	Dysphoric mood	Unstable mood	Do you ever cry about nothing?	96	CAPE
General	Dysphoric mood	Unstable mood	Has your mood been depressed, sad, melancholic, subdued or desperate over weeks?	97	ERIraros
General	Motor disturbance	New movement: stereotypes, echopraxia	Nervous movements or twitching	98	CBCL
General	Motor disturbance	Loss of automatic skills	I simply forgot many of my habits.	99	YSR
General	Motor disturbance	Compulsive motor rituals	I repeat certain acts over and over	100	YSR
General	Motor disturbance	Compulsive motor rituals	I pick my skin or other parts of my body	101	YSR
General	Motor disturbance	Dyskinetic movements of head, face, extremities	Parts of my body twitch or make nervous movements	102	PQ
General	Sleep disturbance	Sleep disturbance	I have trouble sleeping	103	PQ
General	Sleep disturbance	Sleep disturbance	I have not been sleeping well	104	PQ
General	Sleep disturbance	Sleep disturbance	Sleep less than most kids	105	CBCL

General	Sleep disturbance	Sleep disturbance	Have you had any sleep problems (difficulties falling asleep, sleeping through the night, early wakening)? Has your appetite or sexual interest deteriorated?	106	ERIraros
General	Impaired tolerance to normal stress	Exhausted by stressful situations	Everyday things have been more stressful than before, like school or work, social situations, deadlines or changes in a schedule	107	PQ
General	Impaired tolerance to normal stress	Exhausted by stressful situations	Lower level of resilience	108	Self-screen Prodrome
General	Impaired tolerance to normal stress	Exhausted by stressful situations	Increased sensitivity, more easily moved	109	Self-screen Prodrome
General	Impaired tolerance to normal stress	Exhausted by stressful situations	Everyday things have affected me more than they used to	110	PQ
Positive symptoms	Perceptual abnormalities/ hallucinations	Pseudo hallucinations	My thoughts have been so strong that I could almost hear them	111	PQ
Positive symptoms	Perceptual abnormalities/ hallucinations	Pseudo hallucinations	I have noticed strange feelings on or just beneath my skin, like bugs crawling	112	PQ
Positive symptoms	Perceptual abnormalities/ hallucinations	Pseudo hallucinations	I have mistaken shadows for people or noises for voices	113	PQ
Positive symptoms	Perceptual abnormalities/ hallucinations	Unusual perceptual experience	I have seen unusual things like flashes, flames, blinding light, or geometric figures	114	PQ
Positive symptoms	Perceptual abnormalities/ hallucinations	Unusual perceptual experience	Changes in perception (e.g., hearing, seeing, smelling or tasting unusual things)	115	Self-screen prodrome
Positive symptoms	Perceptual abnormalities/ hallucinations	Unusual perceptual experience	Do things sometimes seem fragmented to you, like a photograph that has been torn into pieces and then glued back together?	116	PCA
Positive symptoms	Perceptual abnormalities/ hallucinations	Frank hallucinations	Do you see things that others can't or don't see?	117	YPARQ-B
Positive symptoms	Perceptual abnormalities/ hallucinations	Frank hallucinations	Do you ever hear sounds that are not there?	118	YPARQ-B
Positive symptoms	Perceptual abnormalities/ hallucinations	Frank hallucinations	Do you ever see objects, people or animals that other people cannot see?	119	CAPE
Positive symptoms	Suspiciousness/ persecutory ideas	Suspiciousness or paranoid thinking	I have been concerned that my closest friends and co-	120	PQ

			workers were not really loyal or trustworthy		
Positive symptoms	Suspiciousness/ persecutory ideas	Suspiciousness or paranoid thinking	Feeling people are watching you or giving you a hard time for no reason	121	EDPCC
Positive symptoms	Suspiciousness/ persecutory ideas	Suspiciousness or paranoid thinking	Do you ever feel people are plotting against you or planning to harm you?	122	YPARQ-B
Positive symptoms	Suspiciousness/ persecutory ideas	Persecutory ideas of reference	I have felt that other people were watching me or talking about me	123	PQ
Positive symptoms	Suspiciousness/ persecutory ideas	Persecutory ideas of reference	Do you ever feel as if there is a conspiracy against you?	124	CAPE
Positive symptoms	Suspiciousness/ persecutory ideas	Persecutory ideas of reference	"I have thought that other people had it in for me	125	PQ
Positive symptoms	Suspiciousness/ persecutory ideas	Delusional conviction	I sometimes become concerned about the loyalty and trustworthiness of friends or coworkers (duplicated)	126	BSQSP
Positive symptoms	Disorganised communication	Confused, muddled, racing, or slowed down speech	I have had difficulty organizing my thoughts or finding the right words	127	PQ
Positive symptoms	Disorganised communication	Confused, muddled, racing, or slowed down speech	People have found it hard to understand what I say	128	PQ
Positive symptoms	Disorganised communication	Confused, muddled, racing, or slowed down speech	My thinking has felt confused, muddled, or disturbed in some way*	129	PQ
Positive symptoms	Disorganised communication	Loosening or paralysis of associations	Other people have mentioned changes in the way I speak (e.g., my speech has become difficult to understand)	130	Self-screen Prodrome & PQ
Positive symptoms	Disorganised communication	Odd speech	I have used words in unusual ways	131	PQ
Positive symptoms	Disorganised communication	Circumstantial tangential or paralogical speech	I have wondered off the topic or rambled on too much when I was speaking	132	YSR
Positive symptoms	Grandiose ideas	Exaggerated self-opinion	Do you ever feel that you are a very special or unusual person?	133	CAPE
Positive symptoms	Grandiose ideas	Exaggerated self-opinion	I have thought that I am very important or have abilities that are out of the ordinary	134	PS-R
Positive symptoms	Grandiose ideas	Exaggerated self-opinion	Feeling euphoric or especially competent and important.	135	PROD-screen

Positive symptoms	Grandiose ideas	Clear-cut grandiose delusions	I believe that I have special natural or supernatural gifts beyond my talents and natural strengths	136	PS-R
Positive symptoms	Grandiose ideas	Clear-cut grandiose delusions	Do you feel you have unusual healing abilities or powers?	137	YPARQ-B
Positive symptoms	Grandiose ideas	Unusual ideas	I have felt that I didn't exist, the world didn't exist, or that I was dead	138	PQ
Positive symptoms	Grandiose ideas	Unusual ideas	For moments I got the feeling that my body is deformed.	139	ESI
Positive symptoms	Grandiose ideas	Unusual ideas	Have you been repeatedly pondering on certain things (violent or sexual in content or concerning changes in your body)?	140	ERIraros
Positive symptoms	Grandiose ideas	Overvalued beliefs	Have you been preoccupied with unusual, secretive or supernatural things or ideas (e.g. Religious or esoteric in type)?	141	ERIraros
Positive symptoms	Grandiose ideas	Overvalued beliefs	Do you hold beliefs that others would find unusual or different or bizarre?	142	YPARQ-B
Positive symptoms	Grandiose ideas	Overvalued beliefs	Do you believe in the power of witchcraft, voodoo or the occult?	143	CAPE
Positive symptoms	Grandiose ideas	Non-persecutory ideas of reference	People have dropped hints about me or said things with a double meaning	144	PQ
Positive symptoms	Grandiose ideas	Non-persecutory ideas of reference	Feeling that every day things have a special meaning just for you	145	EDPCC
Positive symptoms	Grandiose ideas	Non-persecutory ideas of reference	Feeling that events in the environment or other people’s behaviour specifically concerns oneself	146	Self-screen Prodrome
Positive symptoms	Grandiose ideas	Perplexity and delusional mood	I have confused whether something I experienced was real or imaginary	147	PQ
Positive symptoms	Grandiose ideas	Perplexity and delusional mood	Do familial surroundings sometimes seem unreal to you?	148	YPARQ-B
Positive symptoms	Grandiose ideas	Perplexity and delusional mood	Often I have a feeling that something strange and unusual is happening around me.	149	ESI
Positive symptoms	Grandiose ideas	First rank phenomenology	Do you ever feel as if the thoughts in your head are not your own?	150	CAPE

Positive symptoms	Grandiose ideas	First rank phenomenology	Have your thoughts ever been so vivid that you were worried other people would hear them?	151	CAPE
Positive symptoms	Grandiose ideas	First rank phenomenology	I have felt that I was not in control of my own ideas or thoughts	152	PQ
Cyclothymia	CHARMS	High or low mood	I seem to be a person whose mood goes up and down easily.	153	HPS
Cyclothymia	CHARMS	High or low mood	Have you had periods lasting several days or more when you felt depressed or irritable, and then other periods of several days or more when you felt extremely high, elated, and overflowing with energy?	154	GBI
Cyclothymia	CHARMS	High or low mood	The mood often changes for no reason	155	TEMPS-A
Cyclothymia	CHARMS	Decreased need for sleep	There have often been times when I had such an excess of energy that I felt little need to sleep at night.	156	HPS
Cyclothymia	CHARMS	Decreased need for sleep	I have often been so excited about an involving project that I didn’t care about eating or sleeping.	157	HPS
Cyclothymia	CHARMS	Decreased need for sleep	Have there been times of several days or more when you did not feel the need for sleep and were able to stay awake and alert for much longer than usual because you were full of energy	158	GBI
Cyclothymia	CHARMS	Increased energy	I am considered to be a kind of ‘Hyper’ person.	159	HPS
Cyclothymia	CHARMS	Increased energy	You had more energy than usual	160	MDQ
Cyclothymia	CHARMS	Increased energy	I often have moods where I feel so energetic and optimistic that I feel I could outperform almost anyone at anything*	161	HPS
Cyclothymia	CHARMS	Inflated self-esteem or grandiosity	I seem to have an uncommon ability to persuade and inspire others.	162	HPS
Cyclothymia	CHARMS	Inflated self-esteem or grandiosity	A hundred years after I'm dead, my achievements will probably have been forgotten.	163	HPS
Cyclothymia	CHARMS	Inflated self-esteem or grandiosity	I often get into moods where I feel like many of the rules of life don’t apply to me.	164	HPS

Cyclothymia	CHARMS	Increased goal directed activity	I do most of my work during brief periods of intense inspiration.	165	HPS
Cyclothymia	CHARMS	Increased goal directed activity	I plan more activities or projects	166	HCL-32
Cyclothymia	CHARMS	Increased goal directed activity	You were more active or did many more things than usual	167	MDQ
Cyclothymia	CHARMS	Restlessness	There are times when l am so restless that it is impossible for me to sit still.	168	HPS
Cyclothymia	CHARMS	Restlessness	Have you experienced periods of several days or more when you were feeling down and depressed, and you also were physically restless, unable to sit still, and had to keep moving or jumping from one activity to another?	169	GBI
Cyclothymia	CHARMS	Restlessness	Have you experienced periods of several days or more when, although you were feeling unusually happy and intensely energetic (clearly more than your usual self), you were also physically restless, unable to sit still, and have to keep moving or jumping from one activity to another?	170	GBI
Cyclothymia	CHARMS	More talkative than usual or pressure to keep talking	In unfamiliar surroundings I am often so assertive and sociable that I surprise myself.	171	HPS
Cyclothymia	CHARMS	More talkative than usual or pressure to keep talking	I often get into excited moods where it’s almost impossible for me to stop talking.	172	HPS
Cyclothymia	CHARMS	More talkative than usual or pressure to keep talking	I talk more	173	HCL-32
Cyclothymia	CHARMS	Unusual ideas, clear thinking	I frequently write down the thoughts and insights that come to me when I am thinking especially creatively.	174	HPS
Cyclothymia	CHARMS	Unusual ideas, clear thinking	Have you had periods of extreme happiness and high energy lasting several days or more when what you saw, heard, smelled, tasted, or touched seemed vivid or intense?	175	GBI

Cyclothymia	CHARMS	Unusual ideas, clear thinking	Have you found yourself at times feeling fearful or suspicious of your environment or people around you?	176	GBI
Cyclothymia	CHARMS	Troublesome behaviour	Spending money or get you or your family in trouble	177	MDQ
Cyclothymia	CHARMS	Troublesome behaviour	I take more risks in my daily life, work, or other activities*	178	HCL-32
Cyclothymia	CHARMS	Troublesome behaviour	Have there been times when you have done things – like perhaps driving recklessly, taking a trip on the spur of the moment, creating a public disturbance, being more sexually active than usual, getting into fights, destroying property, or getting into trouble with the law – which you later thought showed poor judgment?	179	GBI
Hypomania	SCID-5 (Attenuated symptoms)	Elevated, expansive or irritable mood	I often feel excited and happy for no apparent reason.	180	HPS
Hypomania	SCID-5 (Attenuated symptoms)	Elevated, expansive or irritable mood	I often have moods where I feel so energetic and optimistic that I feel I could outperform almost anyone at anything*	181	HPS
Hypomania	SCID-5 (Attenuated symptoms)	Elevated, expansive or irritable mood	Have there been periods when, although you were feeling unusually happy and intensely energetic, almost everything got on your nerves and made you irritable or angry (other than related to the menstrual cycle?)	182	GBI
Hypomania	SCID-5 (Attenuated symptoms)	Flight of ideas	I frequently find that my thoughts are racing.	183	HPS
Hypomania	SCID-5 (Attenuated symptoms)	Flight of ideas	Thoughts raced through your head or you could not slow your mind down?	184	MDQ
Hypomania	SCID-5 (Attenuated symptoms)	Flight of ideas	Some ideas and insights come to me so fast I cannot express them all.	185	HPS
Hypomania	SCID-5 (Attenuated symptoms)	Distractibility	I am frequently in such high spirts that I can't concentrate on any one thing for too long.	186	HPS
Hypomania	SCID-5 (Attenuated symptoms)	Distractibility	You were so easily distracted by things around you that you had trouble concentrating or staying on track	187	MDQ

Hypomania	SCID-5 (Attenuated symptoms)	Distractibility	Has there been a period of several days or more when you could not keep your attention on any one thing for more than a few seconds, and you mind jumped rapidly from one thought to another or to things around you.	188	GBI
Borderline personality	SCID-5 (Attenuated symptoms)	Real effort to avoid abandonment (real or imagination)	I am helpless when I'm left on my own.	189	PBQ
Borderline personality	SCID-5 (Attenuated symptoms)	Real effort to avoid abandonment (real or imagination)	Did you in the last three months ever become desperate when you thought that someone you care about was going to abandon you ?	190	BPDSI
Borderline personality	SCID-5 (Attenuated symptoms)	Real effort to avoid abandonment (real or imagination)	Have you made desperate efforts to avoid feeling abandoned or being abandoned (e.g., repeatedly called someone to reassure yourself that he or she still cared, begged them not to leave you, clung to them physically)?	191	MSI-BPD
Borderline personality	SCID-5 (Attenuated symptoms)	Unstable/ intense interpersonal relationship (idealisation or devaluation)	I can really like someone a lot, and then completely lose interest in them.	192	TEMPS-A
Borderline personality	SCID-5 (Attenuated symptoms)	Unstable/ intense interpersonal relationship (idealisation or devaluation)	Were there moments in the last three months at which you thought that your partner was everything you wanted and other moments at which you thought he/she was awful?	193	BPDSI
Borderline personality	SCID-5 (Attenuated symptoms)	Identity disturbance (unstable self-image or sense of self)	Were you in the last three months in diverse situations or with various people so different that you didn't always behave as the same person and that you didn't know anymore who you truly were?	194	BPDSI
Borderline personality	SCID-5 (Attenuated symptoms)	Identity disturbance (unstable self-image or sense of self)	On one day I might have one opinion of myself and on another day I might have a different opinion.	195	SCS

Borderline personality	SCID-5 (Attenuated symptoms)	Identity disturbance (unstable self-image or sense of self)	Have you often felt that you had no idea of who you are or that you have no identity?	196	MSI-BPD
Borderline personality	SCID-5 (Attenuated symptoms)	Impulsivity in at least 2 areas that are potentially self-damaging (not include self harm or suicidal behaviour)	I take more risks in my daily life, work, or other activities*	197	HCL-32
Borderline personality	SCID-5 (Attenuated symptoms)	Impulsivity in at least 2 areas that are potentially self-damaging (not include self harm or suicidal behaviour)	Have you had at least two other problems with impulsivity (e.g. Eating binges and spending sprees, drinking too much and verbal outbursts)?	198	MSI-BPD
Borderline personality	SCID-5 (Attenuated symptoms)	Impulsivity in at least 2 areas that are potentially self-damaging (not include self harm or suicidal behaviour)	How often in the last three months did you irresponsibly spend money and/or spend more money than you actually can spend? (e.g. Gambling, impulsive buying, making many and long phone calls)	199	BPDSI
Borderline personality	SCID-5 (Attenuated symptoms)	Recurrent suicidal behaviour, gestures, or threats, or self-mutilating behaviour	Have you ever thought about or attempted to kill yourself?	200	SBQ-R
Borderline personality	SCID-5 (Attenuated symptoms)	Recurrent suicidal behaviour, gestures, or threats, or self-mutilating behaviour	I thought of hurting myself/ I want to punish myself	201	BSL-23
Borderline personality	SCID-5 (Attenuated symptoms)	Recurrent suicidal behaviour, gestures, or threats, or self-mutilating behaviour	Have you deliberately hurt yourself physically (e.g. Punched yourself, cut yourself, burned yourself)? How about made a suicide attempt?	202	MSI-BPD

Borderline personality	SCID-5 (Attenuated symptoms)	Affective instability due to a marked reactivity of mood-intense feelings that can last from a few hours to a few days	My mood rapidly cycled in terms of anxiety, anger, and depression	203	BSL-23
Borderline personality	SCID-5 (Attenuated symptoms)	Affective instability due to a marked reactivity of mood-intense feelings that can last from a few hours to a few days	When something bad happens, my mood changes very quickly. People tell me I have a very short fuse.	204	ERS
Borderline personality	SCID-5 (Attenuated symptoms)	Affective instability due to a marked reactivity of mood-intense feelings that can last from a few hours to a few days	Unpleasant feelings will escalate and get out of control.	205	PBQ
Borderline personality	SCID-5 (Attenuated symptoms)	Chronic feelings of emptiness	How often in the last three months did you feel bored or empty inside	206	BPDSI
Borderline personality	SCID-5 (Attenuated symptoms)	Chronic feelings of emptiness	Everything seemed senseless to me	207	BSL-23
Borderline personality	SCID-5 (Attenuated symptoms)	Chronic feelings of emptiness	Have you chronically felt empty?	208	MSI-BPD
Borderline personality	SCID-5 (Attenuated symptoms)	Inappropriate intense anger or difficult controlling anger	I get so angry, I feel like I might lose control	209	MAI
Borderline personality	SCID-5 (Attenuated symptoms)	Inappropriate intense anger or difficult controlling anger	I have trouble controlling my temper.	210	BPAQ
Borderline personality	SCID-5 (Attenuated symptoms)	Inappropriate intense anger or difficult controlling anger	How often in the last three months did you swear, scream and/or slam doors	211	BPDSI
Borderline personality	SCID-5 (Attenuated symptoms)	Transient, stress-related paranoia ideas or severe	I have to be on guard at all times.	212	PBQ

		dissociative symptoms			
Borderline personality	SCID-5 (Attenuated symptoms)	Transient, stress-related paranoia ideas or severe dissociative symptoms	Have you often been distrustful of other people?	213	MSI-BPD
Borderline personality	SCID-5 (Attenuated symptoms)	Transient, stress-related paranoia ideas or severe dissociative symptoms	Have you frequently felt unreal or as if things around you were unreal?	214	MSI-BPD

BPAQ = Buss Perry Aggression Questionnaire (Buss & Perry, 1992); BPDSI = Borderline Personality Disorder Severity Index (Arntz et al., 2003); BSL-23 = Borderline Symptom List (Bohus et al., 2007); BSQSP = Brief Self-Report Questionnaire for Screening Putative Pre-Psychotic States (Liu et al., 2013); CAPE = Community Assessment of Psychic Experiences (Stefanis et al., 2002); CBCL = Child Behavior Checklist (Achenbach & Rescorla, 2001); CPRQ = Composite Psychosis Risk Questionnaire (Loewy et al., 2005); EDPCC = Early Detection Primary Care Checklist (Fusar-Poli et al., 2015); ERIraos = Early Recognition Inventory (Rausch et al., 2013); ERS = Emotion Reactivity Scale (Nock et al., 2008); ESI = Eppendorf Schizophrenia Inventory (Niessen et al., 2010); GBI = General Behavior Inventory (Depue et al., 1981); HCL-32 = Hypomania Checklist-32 (Angst et al., 2005); HPS = Hypomanic Personality Scale (Eckblad & Chapman, 1986); MAI = Multidimensional Anger Inventory (Siegel, 1986); MDQ = Mood Disorder Questionnaire (Hirschfeld et al., 2000); MSI-BPD = McLean Screening Instrument for Borderline Personality Disorder (Zanarini et al., 2003); PCA = Perceptual and Cognitive Aberrations Scale (Chapman & Chapman., 1978); PS-R = PRIME Screen – Revised (Miller et al., 2003); PQ = Prodromal Questionnaire (Loewy et al., 2005); SBQ = Suicidal Behavior Questionnaire Revised (Osman et al., 2001); SCS = Self-Concept Clarity Scale (Campbell et al., 1996); TEMPS-A = Temperament Evaluation of Memphis, Pisa, Paris and San Diego – Autoquestionnaire (Akiskal et al., 2005); YPARQ-B = Abbreviated Youth Psychosis At-Risk Questionnaire (Ord et al., 2004); YSR = Youth Self-Report (Achenbach & Rescorla, 2001).

3. ***Additional statistical details***

Software

All analyses were conducted using R (version 4.4.3). Exploratory graph analysis (EGA) was performed with the *EGAnet* package (version 2.3.0), and confirmatory factor analysis (CFA) was executed using *lavaan* (version 0.6-19). For the standard factor model, composite reliability (CR) and average variance extracted (AVE) were calculated using the *psych* package (version 2.5.3), while bifactor indices such as ω_h, ECV, PUC, H, and FD were computed using *BifactorIndicesCalculator* (version 0.2.2). Visualisations of the bifactor structures were generated with *ggplot2* (version 3.5.2). All other analyses were conducted using SPSS version 29.0.2.

EFA

This method estimates a partial correlation network, wherein items are represented as nodes, and edges denote partial correlations between items. Clusters of items (termed "communities") are detected based on their tendency to exhibit stronger interconnections relative to the rest of the network. In the present analysis, a polychoric correlation matrix was used to accommodate the ordinal nature of the data. To evaluate the stability of the identified structure, a non-parametric bootstrap procedure with 2,000 resamples was conducted. Two indices were examined: structural consistency, reflecting how often the same item composition for each dimension was replicated (range 0–1); and item stability, reflecting how often an item was assigned to the same dimension across resamples (range 0–1). Items with stability values below 0.60 were considered unstable and were excluded from subsequent analyses (see Christensen & Gonglio, 2021).

CFA

In the standard model, each latent factor was specified to load onto its corresponding set of items. In the bifactor model, all items loaded onto a general factor in addition to their specific factors, with the general and specific factors specified as orthogonal. To facilitate interpretation, factor variances were fixed at 1, allowing item loadings to be freely estimated. Given the ordinal and non-normal distribution of the data, the Weighted Least Squares Mean and Variance adjusted (WLSMV) estimator was used for both models, in line with best practices for handling ordinal data in CFA. Model fit was evaluated using commonly recommended indices. The chi-square test was reported for descriptive purposes, while recognising its sensitivity to large sample sizes. Values of the Comparative Fit Index (CFI) and Tucker Lewis Index (TLI) $\geq$

0.90 were interpreted as indicating acceptable fit. Similarly, values of the Root Mean Square Error of Approximation (RMSEA) and the Standardised Root Mean Square Residual (SRMR) below 0.08 were considered indicative of acceptable fit. Given the ordinal and non-normally distributed nature of the data, scaled CFI and TLI, along with robust RMSEA and SRMR estimates, were reported for comparative purposes.

Bifactor analysis

For the bifactor model, analyses primarily focused on assessing the influence of the general factor and the reliability of the structure. Specifically, the Explained Common Variance (ECV) was estimated to reflect the proportion of variance attributable to the general factor, while the Percent of Uncontaminated Correlations (PUC) indicated the extent to which item correlations were explained by the general dimension. Overall reliability was assessed using omega total (ω), whereas omega hierarchical (ω_h) estimated the proportion of variance in total scores accounted for by the general factor. Construct replicability (H) was evaluated to determine the likelihood of replicating the latent constructs across independent studies, and factor determinacy (FD) reflected the correlation between estimated and true factor scores. Recommended thresholds suggested that values of ECV $\geq$ 0.70, $\omega_h \geq$ 0.80, H $\geq$ 0.70, and FD $\geq$ 0.90 indicate strong saturation of the general factor, supporting essential unidimensionality (Rodriguez et al., 2016).

Supplementary Materials References

Achenbach, T. M., & Rescorla, L. A. (2001). *Manual for the ASEBA school-age forms & profiles*. University of Vermont, Research Center for Children, Youth, & Families.

Akiskal, H. S., Akiskal, K. K., Haykal, R. F., Manning, J. S., & Connor, P. D. (2005). TEMPS-A: Progress towards validation of a self-rated clinical version of the Temperament Evaluation of the Memphis, Pisa, Paris, and San Diego Autoquestionnaire. *Journal of Affective Disorders, 85*(1–2), 3–16.

Angst, J., Adolfsson, R., Benazzi, F., Gamma, A., Hantouche, E., Meyer, T. D., … Young, A. H. (2005). The HCL-32: Towards a self-assessment tool for hypomanic symptoms in outpatients. *Journal of Affective Disorders, 88*(2), 217–233.

Arntz, A., van den Hoorn, M., Cornelis, J., Verheul, R., van den Bosch, W. M., & de Bie, A. J. (2003). Reliability and validity of the Borderline Personality Disorder Severity Index. *Journal of Personality Disorders, 17*(1), 45–59.

Bohus, M., Kleindienst, N., Limberger, M. F., Stieglitz, R. D., Domsalla, M., Chapman, A. L., … Wolf, M. (2007). The short version of the Borderline Symptom List (BSL-23): Development and initial data on psychometric properties. *Psychopathology, 42*(1), 32–39.

Buss, A. H., & Perry, M. (1992). The Aggression Questionnaire. *Journal of Personality and Social Psychology, 63*(3), 452–459.

Campbell, J. D., Trapnell, P. D., Heine, S. J., Katz, I. M., Lavallee, L. F., & Lehman, D. R. (1996). Self-concept clarity: Measurement, personality correlates, and cultural boundaries. *Journal of Personality and Social Psychology, 70*(1), 141–156.

Chapman, L. J., Chapman, J. P., & Raulin, M. L. (1978). Body-image aberration in schizophrenia. *Journal of Abnormal Psychology, 87*(4), 399–407.

Christensen, A. P., & Golino, H. (2021). Estimating the stability of psychological dimensions via bootstrap exploratory graph analysis. *Psych, 3*(3), 479–500.

Depue, R. A., Krauss, S., Spoont, M. R., & Arbisi, P. (1981). General behavior inventory identification of unipolar and bipolar affective conditions in a nonclinical university population. *Journal of Abnormal Psychology, 98*(2), 117–126.

Eckblad, M., & Chapman, L. J. (1986). Development and validation of a scale for hypomanic personality. *Journal of Abnormal Psychology, 95*(3), 214–222.

Fusar-Poli, P., Raballo, A., & Parnas, J. (2015). What is an attenuated psychotic symptom? On the importance of the context. *Schizophrenia Bulletin, 43*(4), 687–692.

Hirschfeld, R. M. A., Williams, J. B. W., Spitzer, R. L., Calabrese, J. R., Flynn, L., Keck, P. E., … Zajecka, J. (2000). Development and validation of the Mood Disorder Questionnaire. *American Journal of Psychiatry, 157*(11), 1873–1875.

Liu, C. C., Tien, Y. J., Chen, C. H., Chiu, Y. N., Chien, Y. L., Hsieh, M. H., … Hwu, H. G. (2013). Development of a brief self-report questionnaire for screening putative pre-psychotic states. *Schizophrenia Research, 143*(1), 32–37.

Loewy, R. L., Bearden, C. E., Johnson, J. K., Raine, A., & Cannon, T. D. (2005). The Prodromal Questionnaire (PQ): Preliminary validation of a self-report screening measure for prodromal and psychotic syndromes. *Schizophrenia Research, 79*(1), 117–125.

Miller, T. J., McGlashan, T. H., Rosen, J. L., Cadenhead, K., Ventura, J., McFarlane, W., … Woods, S. W. (2003). Prodromal assessment with the structured interview for prodromal syndromes and the scale of prodromal symptoms. *Schizophrenia Bulletin, 29*(4), 703–715.

Niessen, M. A., Dingemans, P. M., van de Fliert, R., Becker, H. E., Nieman, D. H., & Linszen, D. (2010). Diagnostic validity of the Eppendorf Schizophrenia Inventory (ESI). *Psychological Assessment, 22*(4), 935–944.

Nock, M. K., Wedig, M. M., Holmberg, E. B., & Hooley, J. M. (2008). The Emotion Reactivity Scale: Development, evaluation, and relation to self-injurious thoughts and behaviors. *Behavior Therapy, 39*(2), 107–116.

Ord, L. M., Myles-Worsley, M., Blailes, F., & Ngiralmau, H. (2004). Screening for prodromal adolescents in an isolated high-risk population. *Schizophrenia Research, 71*(2–3), 507–508.

Osman, A., Bagge, C. L., Gutierrez, P. M., Konick, L. C., Kopper, B. A., & Barrios, F. X. (2001). The Suicidal Behaviors Questionnaire-Revised (SBQ-R): Validation with clinical and nonclinical samples. *Assessment, 8*(4), 443–454.

Rausch, F., Eifler, S., Esser, G., & Schultze-Lutter, F. (2013). The Early Recognition Inventory ERIraos: Preliminary evaluation of an instrument for early recognition of psychosis. *European Psychiatry, 28*(1), 22–29.

Rodriguez, A., Reise, S. P., & Haviland, M. G. (2016). Evaluating bifactor models: Calculating and interpreting statistical indices. *Psychological Methods, 21*(2), 137–150.

Siegel, J. M. (1986). The Multidimensional Anger Inventory. *Journal of Personality and Social Psychology, 51*(1), 191–200.

Stefanis, C. N., Hanssen, M., Smirnis, N. K., Avramopoulos, D. A., Evdokimidis, I. K., Stefanis, C. B., … van Os, J. (2002). Evidence that three dimensions of psychosis have a distribution in the general population. *Psychological Medicine, 32*(2), 347–358.

Zanarini, M. C., Vujanovic, A. A., Parachini, E. A., Boulanger, J. L., Frankenburg, F. R., & Hennen, J. (2003). A screening measure for BPD: The McLean Screening Instrument for Borderline Personality Disorder (MSI-BPD). *Journal of Personality Disorders, 17*(6), 568–573.
